# Personalized individual-based exercise prescriptions are effective in treating depressive symptoms of college students during the COVID-19: A randomized controlled trial in China

**DOI:** 10.3389/fpsyt.2022.1015725

**Published:** 2023-01-09

**Authors:** Yuanhui Zhao, Wenxing Wang, Mengdie Wang, Fang Gao, Chun Hu, Bowen Cui, Wenlang Yu, Hong Ren

**Affiliations:** ^1^School of Sport Science, Beijing Sport University, Beijing, China; ^2^Key Laboratory of the Ministry of Education of Exercise and Physical Fitness, Beijing Sport University, Beijing, China; ^3^China Institute of Sport Science, Beijing, China

**Keywords:** personalized medicine, aerobic exercise, resistance training, COVID-19, depressive symptoms, College student

## Abstract

**Background:**

The COVID-19 pandemic has seriously increased depression prevalence among the public, including Chinese college students. However, many exercise cannot be performed as usual under the stay-at-home order. This study was a 12-week three-arm randomized controlled trial using the intention-to-treat principle, aiming to explore and compare the feasibility and effect of individual-based personalized aerobic-exercise and resistance-training prescriptions on depressive symptoms in college students, and conclude with some recommendations for individual-based exercise prescriptions.

**Methods:**

Eighty-six college students with depressive symptoms were randomized into aerobic-exercise (AE), resistance-training (RT), and wait-list control (WLC) groups. Participants in two experimental groups received 12-week personalized AE and RT prescriptions on their individual situations, respectively. No intervention was implemented on participants in the WLC group. Depressive symptoms and physical activity (PA) were measured by Zung Self-Rating Depression Scale (SDS) and International Physical Activity Questionnaire-Short Form (IPAQ-SF), respectively. All data were collected at the baseline, 4, 8, and 12 weeks, and 4-week post-intervention.

**Results:**

At 12 weeks, 72.09% of depressive participants improved to “normal.” Participants exhibited a statistical reduction in SDS in all 3 groups (*p* < 0.05) at 12 weeks compared to baseline. Follow-up assessments showed no significant increase in SDS at 4-week post-intervention compared to 12 weeks (*p* > 0.05). The independent *t*-test revealed significantly lower SDS in AE and RT group than in WLC group (*p*_AE_ < 0.001 and *p*_RT_ < 0.05) at 4, 8, and 12 weeks, and 4-week post-intervention. Furthermore, the PA of participants (including total PA and intensities) in both experimental groups represented a significant improvement at 4-week post-intervention compared to baseline (*p* < 0.05), while no differences were observed in the PA of participants in the WLC group (*p* > 0.05).

**Conclusion:**

Personalized exercise prescriptions have good feasibility as they can increase adherence to intervention and reduce serious adverse events. Besides, individual-based personalized aerobic-exercise and resistance-training prescriptions result in a similar effect in relieving depressive symptoms and improving physical activity in college students. The individual-based exercise programs performed in 45- to 60- min with progressive moderate-to-vigorous intensity, 3 times/week for at least 12 weeks, may reduce depressive symptoms in college students during the COVID-19.

## 1. Introduction

The ongoing pandemic of SARS-CoV-2 (COVID-19) has affected millions of people worldwide and further increased depression prevalence among the public (1, 2). The widespread and high mortality nature of COVID-19 has seriously affected individuals’ mental health and well-being in China, including college students (3, 4). Higher levels of stress response during such special circumstance could lead to a higher prevalence and incidence of depression (5–7). Identified risk factors for depression during the COVID-19 pandemic included having family members being diagnosed, low level of social support, prior diagnosis of mental health disorders (5). A national survey in 33 universities found the pre-epidemic prevalence of depressive disorders was about 19.9% in China (7). A recent large-scale survey revealed that the prevalence of depression state was 21.1% among Chinese college students during the pandemic (5), and there is growing evidence showing that the COVID-19 pandemic has increased the incidence of depression by approximately 30% in Chinese college students (8, 9). Therefore, preventative strategies are needed to prevent the current trend of the increasing incidence rate of depression (10).

Recent studies found that the increased rate of depression in college students was correlated with decreased physical activity due to the COVID-19 stay-at-home order (11, 12). As there is a general belief that physical activity and exercise have positive effects on depression (13), many researches have confirmed that a bidirectional relationship exists between physical activity, exercise and depression (14, 15). Existing studies explained this relationship from biological and psychosocial mechanisms (16–18), including changes in neuroplasticity, the endocrine system, self-esteem, exercise satisfaction, etc. Exercise intervention has been proven to be effective in improving physical activity and relieving depressive symptoms comparable to common psychological and medical treatments (19). Specifically, long-term, group-based aerobic exercise intervention has been widely acknowledged as an effective approach to reducing depression in college students (20), but under the stay-at-home order, a home-based exercise program which can be performed individually is more preferred than group-based programs. Moreover, resistance training with own body weight and/or small household appliance-assisted resistance training are more convenient than aerobic training, most of which needs to be performed outdoors. In recent years, researchers gradually recognized the potential positive effects of resistance training on depression (21). Resistance training is an essential part of exercise and has numerous health benefits (22). However, few randomized controlled trials compared the effect of aerobic exercise and resistance training on the depressive symptoms of college students. Therefore, this study used aerobic exercise and resistance training as the exercise type. Besides, extroversion and neuroticism in personality traits can affect the susceptibility of individuals to depression (23, 24), so that influence the effect of exercise in treating depression. In order to ensure the effect of exercise on depressive symptoms can be clearly confirmed, controlling participants to have no significant differences in these two factors is necessary.

Compared to pre-designed exercise programs, personalized adjusted exercise prescriptions after the evidence-based program can decrease the rate of adverse and/or extreme responders (25). Furthermore, personalized exercise prescriptions paid more attention to personal preferences and willingness of participants. The content of this exercise is more targeted and flexible than that of the ordinary pre-designed exercise in terms of exercise types, intensity and progression. Evidence has shown that prescribing exercise as an alternative therapy can have a positive effect for multiple chronic diseases, including depression (26). However, researches on prescribing exercise programs for depression mainly focused on disease-induced depression in older adults, such as Parkinson (27) or stroke (28). Considering the high prevalence and incidence rate of depressive symptoms among college students during the COVID-19, personalized home-based individual exercise prescriptions are needed.

The main purpose of this 12-week randomized controlled trial was to compare the effect of personalized individual-based aerobic exercise and resistance training prescriptions on depressive symptoms and physical activity level. We also concluded with some recommendations for individual-based exercise prescriptions which can be performed at home for college students with depressive symptoms during the COVID-19.

We hypothesized that personalized individual-based exercise prescriptions are effective in treating depressive symptoms of college students during the COVID-19. Furthermore, personalized aerobic exercise and resistance training would achieve a similar improvement in depressive symptoms and physical activity.

## 2. Materials and methods

### 2.1. Study design and study participants

This is a 12-week, three-arm, single-blinded, parallel-group, randomized controlled trial (RCT) comparing the effect of aerobic exercise and resistance training prescriptions on depressive symptoms in college students. This study was approved by the Sport Science Experiment Ethics Committee of Beijing Sport University (No. 2020128H), from October 10, 2020, to August 01, 2021, and followed the ethical guidelines set out in the Declaration of Helsinki. The research was conducted at Beijing Sport University, China.

Participants were 157 depressive college students, recruited primarily through campus and online advertisements. The inclusion criteria were as follows: (1) being full-time undergraduate or graduate students aged between 18 and 25 years old; (2) standard score of Zung Self-Rating Depression Scale (SDS) ≥ 53, but not meeting the diagnostic criteria of the Diagnostic and Statistical Manual of Mental Disorders, 4th Edition (DSM-IV) for depression (29); (3) being inactive (exercise less than 3 times/week and the total time not exceeding 150 min); (4) not participating in other interventions of similar type. The exclusion criteria included: (1) undergoing depression treatment during the prior year; (2) having a psychiatric history or somatic disease history; and (3) having diseases that may affect exercise. Following an online questionnaire (age, gender, SDS, and regular exercise habits) and telephone screening (DSM-IV, other participation interventions), potential participants attended an introductory meeting during which they would provide written informed consent and complete a medical history checklist. Finally, 86 participants (males: *n* = 25; females: *n* = 61) were enrolled in this trail.

### 2.2. Randomization

**FIGURE 1:**
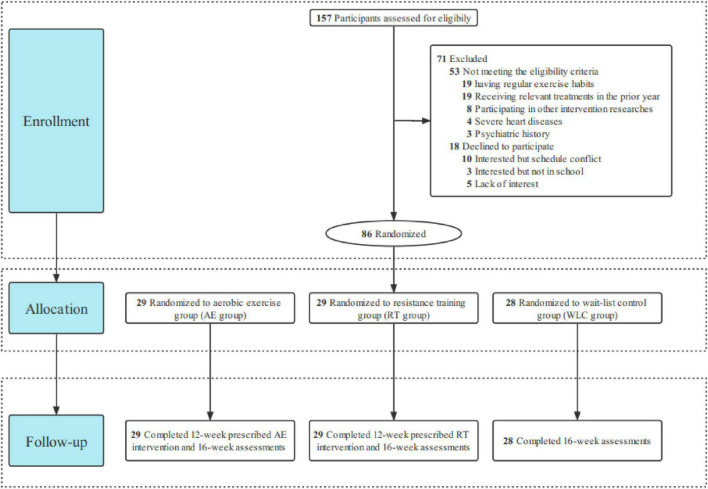
Flowchart.

All eligible participants were randomly allocated to one of the three different groups (aerobic-exercise, resistance training, and wait-list control groups) at a 1:1:1 ratio through computer-based block randomization. The allocation sequence is automatically generated by applying a permuted block design with random blocks of varying length stratified by depressive symptoms and sex. As shown in Figure 1, participants in 2 experimental groups followed 12-week progressive aerobic exercise (AE) or resistance training (RT), respectively, and participants in the wait-list control (WLC) group would receive the exercise intervention after the completion of all assessments. In this research, raters are not allowed to know the randomization process of each participant and conduct intervention sessions, while therapists are not involved in assessing outcomes. Thus, the study strictly adhered to a single-blinded (rater-blinded) design by completely separating intervention and assessment.

### 2.3. Phase I: Exercise interventions prescribing

According to these previous studies, including aerobic-exercise and resistance-training ones (20, 27, 30–32), and *ACSM’s Guidelines for Exercise Testing and Prescription, tenth edition* (33), both AE and RT were performed in 40- to 60-min supervised sessions (including 30- to 40- main exercise), 3 times per week for 12 weeks. The specific content of exercise programs was finally structured and revised based on previous evidence, suggestions of sports medicine experts and results of relevant pre-experiments.

#### 2.3.1. Aerobic exercise prescribing

AE was performed as brisk walking and jogging using treadmills. Each AE session consisted of four parts: warm-up, main exercise, cool-down, and stretching (Table 1). The percentage of heart rate reserve (%HRR) was used to measure the exercise intensity. Average prior-intervention resting heart rate (RHR) for 3 consecutive days, assessed by carotid artery measurement (10-s × 6-s), was used to compute %HRR. Following the principle of progressive physical activity, AE intensity was initially set at 50 ∼ 60% of HRR and gradually progressed up to 60 ∼ 75% and 75 ∼ 90% of HRR (33). To assess whether participants met the target exercise intensity, post-exercise heart rate (HR) was measured immediately after performing Main Exercise. The target post-exercise heart rate (PEHR) was calculated using the %HRR formula: %HRR = (HR exercise − RHR)/(HR max − RHR) × 100%, where HR max = 216.6 − 0.84 × age (34). In addition, participants self-reported the RPE using BORG SCALE (35) after the completion of the main exercise completed. After each AE session, participants were also instructed to measure RHR on the following day to determine if there was a fatigue accumulation (no fatigue accumulation: the increase of RHR < 5 beats/min) (36).

**TABLE 1:** Stage I aerobic exercise program.

Program part	Specific content	Target RPE
Warm-up	3-min dynamic stretching and 3-min accelerating brisk walking.	9 ∼ 11
Main exercise	30-min continuous brisk walking or jogging at 50 ∼ 60% of HRR.	12 ∼ 14
Cool-down	4-min gradually decelerating walking combined with a breathing exercise.	9 ∼ 11
Stretching	Stretching exercises of all parts of the body for at least 10 min.	≤8

We used ratings of perceived exertion (RPE), HR and RH to assess whether participants had adapted to exercise intensity. Specifically, if an individual’s PEHR and RPE were below the target range for 3 consecutive sessions, and there was no fatigue accumulation, the individual would progress to the next exercise intensity stage.

#### 2.3.2. Resistance training prescribing

The RT was performed with dumbbells and elastic bands. Similar to the AE group, each RT session consisted of warm-up, main exercise, cool-down and stretching (Table 2). The finalized RT exercise consisted of six upper-limb (bicep curl, lateral raise, shoulder outward rotation, triceps kickback, and bent Y- and TW-shaped stretch) and six lower-limb plus core exercises (X-band walks, clam-like opening and closing, kneeling hip extension, dynamic glute bridge, and wall squat and plank). The initial intensity was set at 20 repetitions maximum (RM). In order to compare the intensity of 2 experimental groups, the RPE was also used for participants in RT group to assess the post-exercise fatigue. To avoid muscle fatigue accumulation, participants were instructed to alternate between upper-limb and lower-limb plus core exercises during the intervention. The total duration of each repetition, which included both concentric and eccentric phases, was approximately 2-4 s. In addition, participants were instructed to exhale on the concentric phase and inhale on the eccentric phase.

**TABLE 2:** Stage I resistance training program.

Program part	Specific contents and training loads	Target RPE
Warm-up	(1) 3-min dynamic stretching. (2) Alternately perform 15-s high leg lifts and 15-s jumping jacks. Three groups with a 10-s interval between each group.	9 ∼ 11
Main exercise	Alternately perform upper-limb and lower-limb plus core exercises. Three groups × 6 exercise actions × 20 repetitions. 30-s intervals between each action and 2-min intervals between each group.	12 ∼ 14
Cool-down	4-min gradually decelerating walking combined with a breathing exercise.	9 ∼ 11
Stretching	Stretching exercises of training parts for at least 10 min.	≤8

Prior to intervention implementation, the researchers conducted a pre-experiment to determine the optimal exercise modalities and training load of the RT session (see Supplementary Appendix for the procedure of pre-experiments for resistance training prescribing). According to the results of the pre-experiment, we can find the relatively weak aspects of participants and increase targeted resistance exercises.

As mentioned above, the RT was progressive in terms of training load or repetitions. If a participant could complete the standard action of the last group for more than two repetitions in two consecutive sessions (37), the RPE remained unchanged or even decreased and there was no exercise fatigue, the participant would progress to the next stage. The criterion of fatigue accumulation was the same as that of AT group.

### 2.4. Phase II: Intervention implementation

For 12 weeks, participants in AE group received a 30-min session of aerobic exercise three times per week, while participants in RT group received resistance training at the same time and frequency. To ensure intervention fidelity, participants from AE and RT groups were invited for in-person supervised exercise sessions. Participants in WLC group did not receive any exercise intervention.

#### 2.4.1. Intervention monitoring

During the first week, participants completed the training session under the demonstration and guidance of the professionals to avoid incorrect action modes. They were also asked to self-report their RPE and measure their post-exercise HR. Professionals would adapt their exercise prescription to achieve the pre-setting target HR and RPE according to participants’ feedback. If there was a conflict between the HR and RPE, RPE was used. After prescribing exercise programs, participants should complete each session independently. In order to simulate the situation of independent home-based exercise during the COVID-19, the researchers only provided necessary exercise guidance to participants. Each exercise session had a maximum of two participants.

To monitor the exercise adaptability of each participant and progress the exercise stage, training logs were used during the intervention. Participants in both experimental groups were required to fill in the training log, including HR and RPE after the main exercises and RHR in the next morning. In addition, researchers would measure the post-exercise HR in the last session of each week. Participants with at least an 80% attendance rate were included in the final sample.

### 2.5. Outcome measures

The primary outcome, depressive symptoms of participants, was measured using the Zung Self-Rating Depression Scale (SDS), which is a self-report questionnaire. The SDS has been widely adopted in clinical research and has reported good reliability and validity in various populations (38–42), including college students (9, 43, 44). This scale consists of 20 items, of which 10 are reverse scoring. Each item is rated on a 4-point scale. The standard score of the SDS ranges from 25 to 100, and a high score represents a high level of depressive symptoms. In the Chinese norm, a score from 53 to 62 indicates mild depression, while a score from 63 to 72 indicates moderate depression and more than 73 indicates severe depression. In this study, the Cronbach’s alpha is 0.829.

Secondary outcomes included (1) sensitivity to intervention and the possibility of exercise adherence as measured by the neuroticism and extraversion subgroups of Neuroticism Extraversion Openness Five Factor Inventory (NEO-FFI) (45, 46); (2) physical activity (PA) as measured by the International Physical Activity Questionnaire-Short Form (IPAQ-SF) (47–49).

The SDS was administered at each time point during the study: baseline (T_0_), 4 weeks (T_1_), 8 weeks (T_2_), 12 weeks (immediately after the intervention) (T_3_), and 16 weeks (4 weeks post-intervention) (T_4_). The IPAQ-SF was assessed at T_0_ and T_4_, while the NEO-FFI was only assessed at T_0_.

### 2.6. Sample size

The prior sample size for this research was calculated by G*Power version 3.1.9.7 (*F*-tests; ANOVA, Repeated measures, within-between interaction) using the following equation:


$$\sqrt{N\sum_{i=1}^{k}c_{i}^{2}/n_{i}}=\frac{\left|\sum_{i=1}^{k}\mu_{i}c_{i}\right|}{f\times \sigma }$$

where *N, n*_*i*_ denote total sample size and sample size in group *i*, respectively, and *k* is the number of levels, *f* is the effect size, σ and *c* represents the standard deviation and weights.

According to a meta-analysis on the effect of aerobic exercise on depression, the effect size of 0.66 was used (32). The other meta-analysis showed the effect size of 0.42 on the effect of resistance training on depression (21). After averaging the 2 effect sizes and assuming an attrition rate of 25%, a sample size of 18 participants with 6 participants per group was required to provide a three-arm trial with 95% power to detect an effect size of at least 0.54 at a 5% level of significance. In this study, a total of 86 samples with depressive symptoms were finally recruited in Beijing Sport University, China.

### 2.7. Statistical analysis

Descriptive statistics were used to summarize the demographics, physical activity and depressive symptoms of the participants at each time point. According to the data type, all values are expressed as mean ± SD, quartile or constituent ratio. The analysis of variance (ANOVA) and Chi-square (χ^2^) test were used to analyze the differences of baseline characteristics, personality traits, PA and depressive symptoms. All participants were examined at each time point for changes in depressive symptoms. The intention-to-treat procedure was used in this research. A two-way analysis of variance with repeated measures (time point as within-subject factor and intervention group as a between-subject factor) was run to examine whether depressive symptoms changed over time in participants across experimental and control groups. A partial eta-squared ($\eta_{\text{p}}^{2}$) value was calculated to estimate effect size. Besides, differences in PA between 3 groups from T_0_ to T_4_ were tested by ANOVA, and differences between different time points in three groups were tested by paired *t*-test. The statistical results would be corrected by the Greenhouse—Geisser method for the degree of freedom and *p*-value. To confirm the data validation, two raters separately input the data and checked it jointly. Statistical analysis was performed using SPSS statistical software, version 18.0 (IBM Corporation). All statistical tests were 2-tailed with a 5% level of statistical significance.

## 3. Results

### 3.1. Baseline characteristics of participants

Experimental and control groups were similar in baseline characteristics (Table 3). The mean (SD) age of participants was 21.20 (2.10) years, and 61 of 86 were females (70.9%). For educational level, masters and doctors were seen in 27 and 3 participants (31.4% and 3.5%), and most participants were undergraduates (65.1%). Of the participants, the mean (SD) of PA was 1370.65 (1410.02) and most individuals (33.72%) preferred to walking. The means (SD) of neuroticism and extraversion scores were 38.19 (7.18) and 24.43 (4.47). For depressive symptoms, the mean (SD) of SDS was 62.48 (6.62). No significant heterogeneity of demographic and clinical baseline characteristics among participants of 3 groups (Table 4, *p*: 0.061 ∼ 0.957 > 0.05).

**TABLE 3:** Baseline characteristics of the participants (*N* = 86).

Characteristic	All (*N* = 86)	Participants, no. (%)			*P*-value
		**AE group (*n* = 29)**	**RT group (*n* = 29)**	**WLC group (*n* = 28)**	
Age, years	21.20 (2.10)	20.72 (2.05)	21.66 (1.97)	21.21 (2.25)	0.243
**Sex**					
Male	25 (29.1)	8 (27.6)	9 (31.0)	8 (28.6)	0.957
Female	61 (70.9)	21 (72.4)	20 (69.0)	20 (71.4)	
**Educational level**					
Undergraduates	56 (65.1)	21 (72.4)	17 (58.6)	18 (64.3)	0.859
Masters	27 (31.4)	7 (24.1)	11 (37.9)	9 (32.1)	
Doctors	3 (3.5)	1 (3.4)	1 (3.4)	1 (3.6)	
**Physical activity, MET-min/week**					
Total	1370.65 (1410.02)	1405.93 (1280.64)	1591.31 (1623.99)	1105.55 (1302.56)	0.429
Walking	561.58 (864.44)	687.31 (824.34)	518.90 (857.32)	475.55 (926.80)	0.624
Moderate intensity	321.16 (534.84)	227.59 (418.31)	374.48 (423.49)	362.86 (720.30)	0.516
Vigorous intensity	487.91 (814.18)	491.03 (704.83)	697.93 (1097.25)	269.14 (474.54)	0.136
**NEO-FFI**					
Neuroticism scores	38.19 (7.18)	38.55 (7.73)	36.59 (7.49)	39.61 (5.94)	0.320
Extraversion scores	24.43 (4.47)	25.38 (3.10)	24.96 (4.47)	22.61 (5.47)	0.061
Depression state-SDS	62.67 (6.43)	62.55 (6.44)	64.48 (5.72)	60.93 (6.81)	0.112

For continuous variables, an analysis of variance was used, and for categorical variables, a χ^2^ test was used to compare differences between three groups.

Data are presented as mean (SD).

**TABLE 4:** The implementation of exercise intervention of the participants.

Variables	AE group (*n* = 29)	RT group (*n* = 29)	WLC group (*n* = 28)	*P*-value
**Duration of stages, weeks**				
Stage I	3.24 (0.95)	3.93 (1.00)	–	0.009**
Stage II	4.86 (1.16)	4.66 (0.90)	–	0.450
Stage III	3.90 (1.52)	3.34 (1.62)	–	0.191
Attendance of exercise interventions, times/%	33.48 (2.25)/93.00 (6.24)	33.17 (2.54)/92.15 (7.04)	–	0.624

For all the variables, an independent *t*-test was used.

**Represents a significant difference between AE group and RT group in time point, **p < 0.01.

Data are presented as mean (SD).

### 3.2. The implementation of exercise intervention

Of 157 potential participants screened, 53 participants did not meet the eligibility criteria and 18 declined to participate. The enrollment rate of this study was 54.78% (Figure 1). Of 86 participants randomized, 2 experimental groups were randomly assigned to 29 participants and the WLC group was assigned to 28. Participants randomized to 2 experimental groups were asked to attend at least 29 sessions (total of 36), while participants randomized to the control group did not attend any exercise interventions. After completing all exercise interventions (Table 4), the mean (SD) attendance rates were 92.58% (6.61%) for all experimental participants, 93.00% (6.24%) for participants in AE group and 92.15% (7.04%) for participants in RT group. Besides, 9 of 29 participants (31.03%) in each group attended all sessions. All the 86 participants completed 16-week measurements with none dropout.

For experimental groups, the average duration of stage I was 3.24 (0.95) weeks for AE group and 3.93 (1.00) weeks for RT group. The independent *t*-test showed a significant difference of durations of stage I between groups. Stage II took the longest durations in both AE [3.90 (1.52) weeks] and RT groups [3.34 (2.54) weeks].

During the intervention, 5 participants (8.47%) of experimental groups self-reported mild knee or ankle pain with exercises (1 from AE and 4 from RT), which resolved with the use of a thick towel or reduce speed. Another common adverse event in the intervention was delayed muscle soreness. Thirty-seven participants (62.71%) reported this event after the first two sessions (17 from AE and 20 from RT). The rates of delayed muscle soreness decreased to 6.78% (1 from AE and 3 from RT) after 2 weeks and 1.69% (1 from RT) after stage I. No other serious adverse events were reported.

### 3.3. Efficacy of interventions on depressive symptoms and PA in college students

**FIGURE 2:**
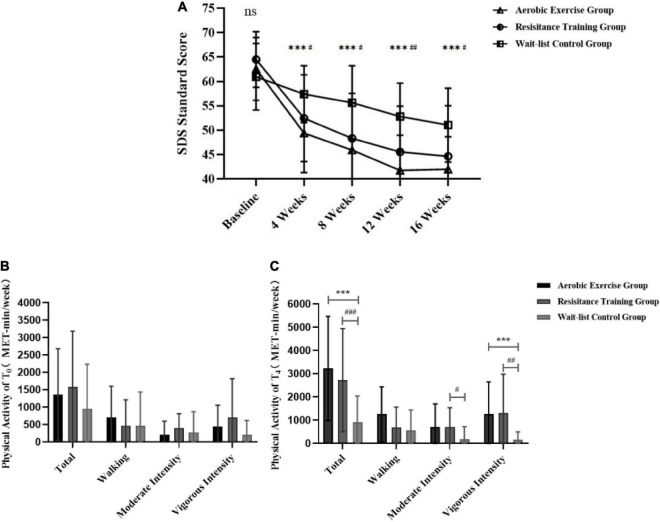
Comparison of between-group differences in depression state and physical activity (continuous) for AE, RT, and WLC groups. **(A)** The between-group differences for SDS standard scores. **(B)** Baseline PA differences by intensity. **(C)** PA intensity differences 4 weeks after intervention. ns Represents no significant difference among the three groups in time point. ^***^Represents a significant difference between the AE group and the WLC grou*p* in time point. ^#^Represents a significant difference between the RT group and the WLC group in time point. ^***^p < 0.001; ^#^*p* < 0.05, ^##^*p* < 0.01, and ^###^*p* < 0.001.

The outcome of analysis of SDS was presented in Table 5 and Figure 2. The 3 × 5 RM-ANOVA revealed a significant time × group interaction for SDS [*F*(5.593,151.012) = 9.569, *p* < 0.001, $\eta_{\text{p}}^{2}$ = 0.262]. Both time and group showed significant main effects for SDS [time: *F*(2.331,62.943) = 104.387, *p* < 0.001, $\eta_{\text{p}}^{2}$ = 0.795; group: *F*(2,54) = 29.270, *p* < 0.001, $\eta_{\text{p}}^{2}$ = 0.520]. The contribution of each factor to SDS was that time > group > interaction [$\eta_{\text{p}}^{2}$_time_ > $\eta_{\text{p}}^{2}$_group_ > $\eta_{\text{p}}^{2}$_interaction_). In experimental groups, followed-up analysis for simple effects showed that compared with the previous point, SDS was significantly lower following AE and RT interventions (*p*_*AE*_ < 0.05, *p*_RT_ < 0.05). Furthermore, no significant difference was found in SDS at T_4_ compared to T_3_. In WLC group, simple effects analysis revealed that the SDS was significantly decreased at T_1_ and T_3_ compared to the previous time-point (*p* < 0.05). Besides, significant differences were found in SDS of AE and RT group compared with WCL group (*p*_AE_ < 0.001, *p*_RT_ < 0.05) at all time-points, but no significant differences were revealed between AE and RT group during the study (*p* > 0.05).

**TABLE 5:** Comparison of within-group changes in outcomes variables (continuous) for AE, RT, and WLC groups.

Variables	AE group		RT group		WLC group	
	**Mean (SD)**	***P*-value**	**Mean (SD)**	***P*-value**	**Mean (SD)**	***P*-value**
**SDS**						
Baseline (T_0_)	62.55 (6.44)		64.48 (5.72)		60.93 (6.81)	
4 weeks (T_1_)	49.38 (8.08)	0.000***	52.45 (8.89)	0.000***	57.36 (5.83)	0.016*
8 weeks (T_2_)	45.90 (9.73)	0.046*	48.29 (9.22)	0.003**	55.61 (7.59)	0.152
12 weeks (T_3_)	41.72 (7.22)	0.002**	45.54 (9.39)	0.016*	52.79 (6.87)	0.028*
16 weeks (T_4_)	41.97 (6.65)	0.812	43.82 (9.62)	0.125	51.04 (7.57)	0.070
**IPAQ – PA (total), MET-min/week**						
Baseline (T_0_)	1402.93 (1280.64)	0.001**	1591.31 (1623.99)	0.001**	1146.50 (1308.88)	0.625
16 weeks (T_4_)	3119.17 (2343.46)		2554.02 (2057.66)		1061.52 (1155.04)	
**IPAQ – walking, MET-min/week**						
Baseline (T_0_)	687.31 (824.34)	0.087	518.90 (857.32)	0.186	493.17 (939.67)	0.232
16 weeks (T_4_)	1128.83 (1107.50)		762.98 (983.12)		631.89 (871.11)	
**IPAQ – moderate, MET-min/week**						
Baseline (T_0_)	227.59 (418.31)	0.017*	374.48 (423.49)	0.014*	376.30 (730.44)	0.066
16 weeks (T_4_)	713.10 (1057.39)		618.62 (651.46)		240.00 (674.46)	
**IPAQ – vigorous, MET-min/week**						
Baseline (T_0_)	491.03 (704.83)	0.005**	697.93 (1097.25)	0.047*	277.04 (480.63)	0.430
16 weeks (T_4_)	1277.24 (1450.51)		1172.41 (1591.84)		189.63 (371.37)	

*Represents a significant difference between this point and the previous point.

Data are presented as mean (SD).

*p < 0.05, **p < 0.01, ***p < 0.001.

Table 5 also revealed the outcome of pairwise comparison of PA. A paired *t*-test to compare PA of participants revealed that the total of PA and different intensities (*p* < 0.05), except walking in 2 experimental groups increased significantly at T_4_ compared to baseline: (*p* > 0.05). However, no significant increase of PA was showed in WLC group (*p* > 0.05).

As shown in Figure 2, the independent *t*-test to compare PA of different groups at T_0_ and T_4_ revealed that there was no significant difference among groups at T_0_ (*p* > 0.05), while significant differences were found in the total and vigorous-intensity of PA between the WLC group and two experimental groups at T_4_ (*p* < 0.01). There was also a significant difference of moderate-intensity of PA between RT group and WLC group (*p* < 0.05). No significant difference between AE group and RT group was found at both T_0_ and T_4_ (*p* > 0.05).

At baseline, moderate depression was seen in 27 of 87 participants (31.4%), and most had mild depression (53, 61.63%). The depression level of participants relieved over time, and 62 participants (72.09%) showed no depressive symptoms after the intervention (AE: 26, 89.66%; RT: 21, 72.41%; and WLC: 15, 53.57%). Furthermore, the rates of individuals with no depressive symptoms in WLC group continued to increase during the 4 weeks after intervention, while the rates of other experimental groups remained unchanged (WLC: 19, 67.86%). The Chi-test revealed that there was a significant difference of rates of moderate depression among three groups at different time points (χ^2^ = 20.880, *p* < 0.01) (Table 6).

**TABLE 6:** Numbers of participants with different depression levels in AE, RT, and WLC groups.

Depression state	Participants, no. (%)				χ^2^, *P*-Value
	**All (*N* = 86)**	**AE group (*n* = 29)**	**RT group (*n* = 29)**	**WLC group (*n* = 28)**	
**Normal**					
Baseline (T_0_)	0 (0.00)	0 (0.00)	0 (0.00)	0 (0.00)	1.220 0.976
12 weeks (T_3_)	62 (72.09)	26 (89.66)	21 (72.41)	15 (53.57)	
16 weeks (T_4_)	67 (77.91)	26 (89.66)	21 (72.41)	19 (67.86)	
**Mild**					
Baseline (T_0_)	53 (61.63)	18 (62.07)	13 (44.83)	22 (78.57)	6.495 0.592
12 weeks (T_3_)	19 (22.09)	3 (10.34)	7 (24.14)	9 (32.14)	
16 weeks (T_4_)	14 (16.28)	3 (10.34)	7 (24.14)	5 (17.86)	
**Moderate**					
Baseline (T_0_)	27 (31.40)	9 (31.03)	14 (48.28)	4 (14.29)	20.880 0.007**
12 weeks (T_3_)	5 (5.81)	0 (0.00)	1 (3.45)	4 (14.29)	
16 weeks (T_4_)	5 (5.81)	0 (0.00)	1 (3.45)	4 (14.29)	
**Severe**					
Baseline (T_0_)	6 (6.98)	2 (6.90)	2 (6.90)	2 (7.14)	–
12 weeks (T_3_)	0 (0.00)	0 (0.00)	0 (0.00)	0 (0.00)	
16 weeks (T_4_)	0 (0.00)	0 (0.00)	0 (0.00)	0 (0.00)	

**Represents a significant difference of numbers of participants of the same depression state at different time-points among AE, RT, and WLC groups.

**p < 0.01.

Data are presented as mean (SD).

## 4. Discussion

We found a statistically and clinically significant reduction in depressive symptoms and improvement in PA among all groups. AE and RT prescriptions achieved an effective reduction in depressive symptoms. Our findings also complemented previous evidence by showing that prescribed AE and RT had a similar effect on depressive symptoms in college students. Prescribed exercise programs can be effectively used on relieving depressive symptoms in college students. For PA, our results showed that AE and RT prescriptions were effective to improve PA, including moderate-, vigorous-intensity of PA and total PA.

We achieved a higher program adherence rate than previous studies as all participants completed the 12-week intervention and relevant measurements (27, 50). There is evidence showing a higher adherence rate was associated with a higher level of program satisfaction (51), indicating that our program was not only effective but also satisfying. A strength of our study is that we included multiple individual testing to ensure participants’ physical states and subjective feelings were closely monitored and well maintained. As suggested by Wackerhage (25), such testing can avoid adverse events and improve exercise satisfaction.

The results of our study found the prescribed aerobic exercise and resistance training have positive effects to relieve depressive symptoms of college students. These findings on the effects of exercise interventions in relieving depressive symptoms are consistent with the conclusion of various previous RCT researches (31, 52, 53) and systematic reviews (20, 21, 32). Besides, such positive effect was well-maintained at 4-week post-intervention in our study, which is consistent with previous studies (54, 55), so it is possible that prescribed exercise continuously contributed to the participants’ abilities to deal with depressive stress. For human survival and adaptation, depressive symptoms are very complex psychological activities, and would be affected by many factors, which can be attributed to individual stress. Previous researches believed the specific impact of stress depended on the method individuals deal with it, and the stress itself didn’t be distinguished between positive and negative ones (56, 57). As Jackson has confirmed, appropriate stress inoculation training can trigger the (over)compensation mechanism of brain, so that the brain can be more well-prepared to deal with following depressive stress (58). Considering the effect of exercise, we believed that both aerobic exercise and resistance training at a certain intensity can be regarded as one form of the stress inoculation, but this hypothesis needs to be verified by further researches. Many other researchers revealed that through exercise intervention, depression can be relieved due to other changes of physiological, psychological and sociological mechanisms (56, 59). Furthermore, future research with a longer follow-up period is needed to ensure the long-lasting effect of personalized exercise prescription on depressive symptoms.

Previous studies found that depression is associated with physical activity of individuals, especially moderate intensity of PA (60). In this study, prescribed aerobic exercise and resistance training both has positive effects in increasing PA (including the amount and intensity) of college students, and such positive effect was well-maintained at 4-week post-intervention. This result is familiar with the *trans*-theoretical model of stage of change (61)— the process of exercise intervention is the period of action, which is more likely to promote individuals to enter the period of maintenance, especially intervention more than 6 months. Moreover, exercise could enable college students to get more satisfaction and enjoyment, and this process would promote them to exercise regularly (62). As many researchers suggested a correlation between low levels of physical activity and symptoms of depression (63–65), improvement in PA of participants from both exercise groups also proved that the potential mediating role of PA on reliving depressive symptoms. To relieve depressive symptoms in college students, we recommend individual-based AE and RT programs, three sessions per week for at least 12 weeks, lasting 45- to 60-min per session. Individuals can self-choose exercise types, such as brisk walking, jogging for aerobic exercise and/or resistance training with household simple exercise equipment. For aerobic exercise, heart rate and RPE can be used to monitor the intensity, and the recommended initial intensity was 50 ∼ 60% HRR and gradually processed to 60 ∼ 90% HRR. RM and RPE can be used to monitor the intensity of resistance training. For college students with no regular exercise habits, some easy-to-learn exercises are recommended. Table 2 presents recommended exercise modes and the corresponding weight of dumbbells and elastic bands. According to the implementation of interventions, the duration of stage I, for both AE and RT programs, could be 4 weeks, and 5 weeks of stage II. Additionally, warm-up, cool-down and stretching exercises are needed in each session. Furthermore, according to the intervention mode and results of this study, we can infer that home-based AE and RT prescriptions are effective in treating depressive symptoms of college students during the COVID-19, but the specific effect still needs further research to confirm.

The strengths of this study include a randomized controlled trial design with personalized exercise program prescribing and training log design to ensure the involvement and adherence of participants, the adaption and safety of exercise intervention for experimental groups, and multiple follow-up time points to elucidate the residual effects of different interventions. However, this research inevitably had limitations which must be considered in interpreting the results. First, expectation bias may exist in this study due to awareness of the treatment allocation. Second, the depressive symptoms was evaluated only by self-reported scales without other objective indexes, such as electroencephalography (EEG) or blood index, to support and analyze the results. Third, this study only simulated the state of home-based exercise, but did not analyze the method that participants can use to keep the exercise routine when they do it themselves. Furthermore, there could be other confounders and covariates, such as unequal numbers of participants of genders and educational levels, that were not included in this study model.

## 5. Conclusion

Among college students with depressive symptoms, personalized aerobic-exercise and resistance-training prescriptions resulted in an effective and similar reduction in depressive symptoms and improvement in physical activity. Exercise types did not play a significant role in their effect on depressive symptoms. Our findings suggest that personalized exercise prescriptions have good feasibility in relieving depressive symptoms in college students. We concluded with recommendations for home-based exercise programs for college students during the COVID-19 which can be adapted by future investigators and practitioners.

## Data availability statement

The raw data supporting the conclusions of this article will be made available by the authors, without undue reservation.

## Ethics statement

The studies involving human participants were reviewed and approved by Sport Science Experiment Ethics Committee of Beijing Sport University. The patients/participants provided their written informed consent to participate in this study.

## Author contributions

YZ and WW designed the study, analyzed the data, and wrote the initial manuscript. FG, BC, CH, and WY recruited the participants and supervised the exercise. HR and MW designed the exercise programs and revised the manuscript critically. All authors participated in drafting the manuscript, read, and approved the final version of the manuscript.
