# Supplementary material for: Personalized individual-based exercise prescriptions are effective in treating depressive symptoms of college students during the COVID-19: A randomized controlled trial in China
Source: Front Psychiatry. 2023 Jan 9;13:1015725. doi: 10.3389/fpsyt.2022.1015725 (PMC9869038; doi:10.3389/fpsyt.2022.1015725)
Supplement: Supplementary file 1 [file Table_1.DOCX]

Supplementary Material

# Supplementary Appendix

**Methods 1: Pre-Experiments for Resistance Training Prescribing**

In the pre-experiments, 16 participants (8 males and 8 females, the same level of physical activity as the participants of this study) were recruited and were asked to complete 3 tests for 3 different loads. The interval of 2 tests was not more than 48 hours. In each test, the participants were required to try their best to complete the maximum time or repetitions of standard exercise actions (**Supplementary Table 1**). According to the results of the pre-experiment, researchers chose the training load which was closest to the setting RM as the initial training load.

Supplementary Table 1. Maximum times or repetitions of each exercise action under different loads (Mean ± SD)

| **Resistance Training Actions** | **Gender** | **Load 1** | **Load 2** | **Load 3** |
| --- | --- | --- | --- | --- |
| Upper-Limb (Dumbbells) (times) |  |  |  |  |
| Bicep Curl | Male | 27.9 ± 4.7 | 24.5 ± 4.8 | 20.9 ± 4.1^*^ |
|  | Female | 24.8 ± 3.9 | 21.0 ± 3.0^*^ | 18.3 ± 3.2 |
| Lateral Raise | Male | 25.4 ± 3.0 | 21.5 ± 2.6^*^ | 17.4 ± 2.1 |
|  | Female | 20.9 ± 2.6^*^ | 17.6 ± 2.9 | 14.5 ± 2.2 |
| Shoulder Outward Rotation | Male | 34.1 ± 5.7 | 29.5 ± 6.0 | 24.3 ± 6.1^*^ |
|  | Female | 29.1 ± 2.9 | 25.4 ± 3.2 | 21.1 ± 2.8^*^ |
| Triceps | Male | 26.8 ± 2.9 | 23.1 ± 3.4 | 19.3 ± 3.7^*^ |
|  | Female | 22.3 ± 2.5 | 19.9 ± 2.7^*^ | 16.0 ± 2.9 |
| Bent Y-Shaped Stretch | Male | 21.6 ± 2.6^*^ | 17.9 ± 2.5 | 13.1 ± 2.5 |
|  | Female | 19.9 ± 2.5^*^ | 16.9 ± 2.6 | 13.5 ± 2.3 |
| Bent TW-Shaped Stretch | Male | 25.4 ± 2.7 | 22.5 ± 3.2 | 18.9 ± 3.3^*^ |
|  | Female | 20.6 ± 2.2^*^ | 17.9 ± 2.0 | 15.0 ± 2.1 |
| Lower-Limb (Elastic Bands) (times) |  |  |  |  |
| X-Band Walks | Male | 30.4 ± 3.4 | 25.3 ± 3.7 | 20.8 ± 3.6^*^ |
|  | Female | 24.1 ± 2.9 | 20.2 ± 2.0^*^ | 16.0 ± 2.3 |
| Clam-Like Opening and Closing | Male | 28.7 ± 3.7 | 24.0 ± 3.4 | 18.3 ± 3.9^*^ |
|  | Female | 30.4 ± 5.0 | 26.6 ± 4.7 | 21.3 ± 4.0^*^ |
| Kneeling Hip Extension | Male | 21.4 ± 2.4^*^ | 17.6 ± 2.8 | 14.3 ± 2.8 |
|  | Female | 26.1 ± 4.6 | 22.0 ± 4.2^*^ | 17.7 ± 3.9 |
| Dynamic Glute Bridge | Male | 29.6 ± 3.0 | 24.8 ± 3.3 | 20.7 ± 3.6^*^ |
|  | Female | 29.0 ± 3.1 | 24.9 ± 3.3 | 20.8 ± 3.4^*^ |
| Core Exercises (Unarmed) |  |  |  |  |
| Wall Squat (s) | Male | 69.0 ± 20.3 | - | - |
|  | Female | 50.5 ± 17.5 | - | - |
| Plank (s) | Male | 80.4 ± 11.3 | - | - |
|  | Female | 52.9 ± 14.4 | - | - |

Load 1: 1kg dumbbells and 15 lb elastic bands for females; 2kg and 20 lb for males.

Load 2: 1.5kg dumbbells and 20 lb elastic bands for females; 3kg and 30 lb for males.

Load 3: 2kg dumbbells and 30 lb elastic bands for females; 4kg and 40 lb for males.

In the unilateral action, only the dominant side was measured.

^*^ The optimal load for corresponding exercise action of stage I RT program.
